# Coherent coupling between Vanadyl Phthalocyanine spin ensemble and microwave photons: towards integration of molecular spin qubits into quantum circuits

**DOI:** 10.1038/s41598-017-13271-w

**Published:** 2017-10-12

**Authors:** C. Bonizzoni, A. Ghirri, M. Atzori, L. Sorace, R. Sessoli, M. Affronte

**Affiliations:** 1 Dipartimento di Scienze Fisiche, Informatiche e Matematiche, Università di Modena e Reggio Emilia, via G. Campi 213/A, 41125 Modena, Italy; 2 Istituto Nanoscienze S3, CNR via G. Campi 213/A, 41125 Modena, Italy; 3 Dipartimento di Chimica “Ugo Schiff” & INSTM RU, Università degli Studi di Firenze, Via della Lastruccia 3, 50019 Sesto Fiorentino (Firenze), Italy

## Abstract

Electron spins are ideal two-level systems that may couple with microwave photons so that, under specific conditions, coherent spin-photon states can be realized. This represents a fundamental step for the transfer and the manipulation of quantum information. Along with spin impurities in solids, molecular spins in concentrated phases have recently shown coherent dynamics under microwave stimuli. Here we show that it is possible to obtain high cooperativity regime between a molecular Vanadyl Phthalocyanine (VOPc) spin ensemble and a high quality factor superconducting YBa_2_Cu_3_O_7_ (YBCO) coplanar resonator at 0.5 K. This demonstrates that molecular spin centers can be successfully integrated in hybrid quantum devices.

## Introduction

Coherent coupling of single photons with a resonant two-level center (TLC), being this either a single atom or a spin, leads to states of the whole system which are not eigenvalues of the single TLC or of the single photon, as described by the Jaynes-Cummings model^1,2^. This condition is achieved when the coupling strength (Ω) between a photon and the TLC is larger than the decay rates of the photons in the cavity (κ) and that of the TLC (γ). The relevant figure of merit is the cooperativity *C*, defined as $C={{\rm{\Omega }}}^{2}/{\rm{\gamma }}{\rm{\kappa }}$. Typical spectroscopic experiments at microwave (MW) frequencies, such as Continuous Wave Electron Spin Resonance (CW-ESR), are performed in the weak coupling regime, in which Ω < γ and *C* < 1. On the other hand, when Ω is high enough to balance the decay rates γ and κ (i.e. Ω ≈ γ with Ω, γ > κ), *C* ≥ 1 and the system enters in a regime of high cooperativity, in which photons are coherently exchanged between the cavity and the TLC system. In the frequency domain, the genuine feature of this quantum behaviour is the presence of both the anti-crossing of the spin-photon eigenstates and of the splitting of the frequency spectrum on resonance (Rabi splitting)^3,4^. Achieving high cooperativity is a fundamental step for developing Quantum Technologies since it allows to coherently transfer quantum information between different solid state registers and flying qubits in hybrid devices^5–7^.

Coherent coupling is typically achieved between atoms and photons in optical cavities or between superconducting quantum devices and microwave resonators through spectroscopic experiments. Although spins are ideal TLCs, their small magnetic dipole moment normally gives very small coupling rates, Ω_S_, (on the order of Hz for a single spin) with the magnetic component of the oscillating field, and obtaining coherent coupling is intrinsically not trivial. A possible way to enhance the spin-photon coupling is to use spin ensembles^3^. In this case, according to the Tavis-Cummings model, collective spin modes couple to a single photon resulting in the enhancement of the single spin coupling by a factor $\sqrt{N}$ (with *N* the number of spins)^8,9^. Thus, by taking *N* ≈ 10^12^–10^16^, the collective coupling rate Ω = Ω_S_ $\sqrt{N}$ may well be larger than MHz. By following this approach, high cooperativity has been reached with Nitrogen Vacancies (NV) centers^10–12^ and Erbium impurities in inorganic crystals^13,14^ coupled to superconducting planar resonators.

As far as molecular spin system are concerned, they have demonstrated to constitute an ideal playground to study quantum phenomena at the nanoscale. Seminal investigations showed that coherent spin manipulation of these systems is possible by using pulsed electron spin resonance spectroscopy^15–18^, while recent investigations have convincingly shown that the decoherence times can be tailored at synthetic level^16,19–30^. This resulted in the observation of Rabi oscillations in ESR nutation experiments even at room temperature^19,21,25^, paving the way for the integration of these systems in hybrid quantum circuits. In particular, the next challenge along this line is to embed molecular spins in a circuit Quantum-Electrodynamics (Circuit-QED) architectures^31–33^. This may provide the possibility to realize hybrid quantum memories^34^ and to investigate the generation of macroscopic entanglement^35^ with molecular spin systems.

Coupling experiments between molecular spins and microwave photons in a 3D cavity have been recently reported^9,36,37^. Moreover, concentrated spin ensembles of organic radicals have been efficiently coupled to planar resonators by exploiting the exchange narrowing of their ESR lines^31,38–40^. Diluted ensembles potentially show longer coherence times but the conditions for reaching the strong coupling regime with these samples still need to be found. This constitutes an important step in the perspective of coupling few – eventually one - molecular spin(s) to superconducting devices^32,33^. To achieve this condition, resonators should have sufficiently high quality factors (*Q* = ν_0_/κ, where *ν*_0_ is the resonant frequency in zero magnetic field) to provide photons with sufficiently long lifetimes.

The aim of this work is to investigate the collective coupling between coherently manipulable molecular spin ensembles and superconducting coplanar resonator. We focus on the recently investigated VOPc molecular spin qubit^25^ as a suitable spin ensemble, which can be diluted in its isostructural TiOPc (Titanyl Phtalocyanine) diamagnetic matrix. This choice is motivated by *i)* the long *T*_1_ (spin-lattice) and *T*_2_ (spin-spin) relaxation times over a large temperature range^25^, (*ii*) the sufficiently sharp lines at resonance frequencies, and *iii)* its potential to be deposited on surfaces and embedded in molecular-scale devices^41,42^. We use high-Tc YBCO superconducting films for the fabrication of the resonators, which allow us to extend the investigation up to 30 K and above^39^. Because the linewidth of the spin ensemble is affected by inhomogeneous broadening, and the total number of involved spins affects the coupling, we use different resonators and investigate different sample concentrations in order to find optimal working conditions. Rabi splitting of the energy spectrum is observed at 0.5 K, indicating the presence of coherent hybrid spin-photon coupling.

## Results

### Samples

**Figure 1:**
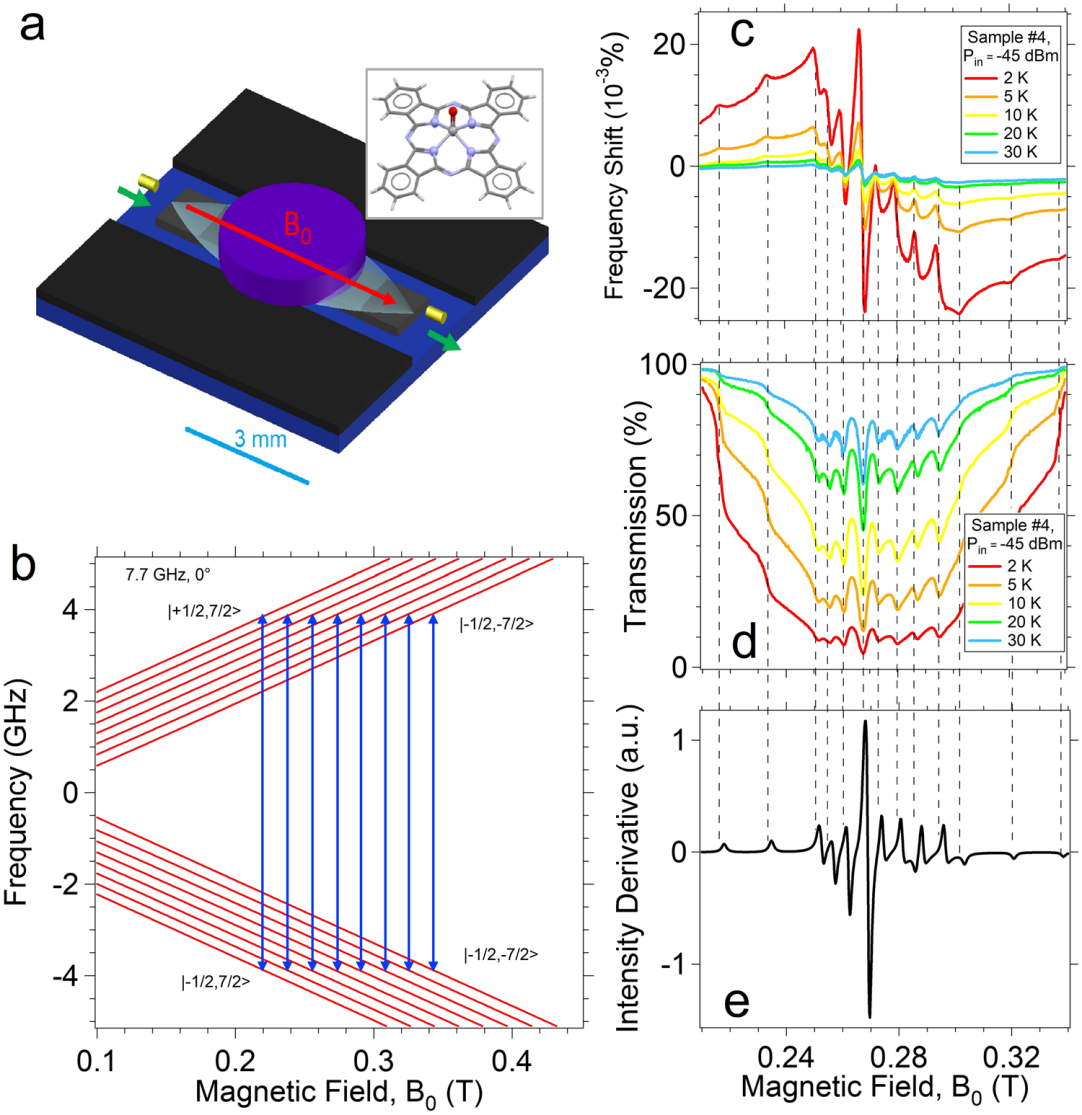
Transmission spectroscopy of VOPc sample #4 in the weak coupling regime at different temperatures. The resonant frequency at zero field is 7.66 GHz (Res #2). (**a**) Sketch of the resonator with a VOPc pellet (purple) on it. Transparent light blue area represents the fundamental resonant MW mode and the green arrows indicate the path of the microwaves. The red arrow shows the direction of the static magnetic field, B_0_. Yellow cylinders represent the antennas for the injection and the collection of the MW signal. The light blue scale bar corresponds to 3 mm. A sketch of the VOPc molecular structure is reported in the inset. (**b**) Easy Spin simulation of the energy levels for a VOPc molecule with the static magnetic field applied along the z direction (i.e along the V = O bond) based on the values reported in^25^. The blue arrows represents the eight |−*m*_S_, *m*_I_> → |-*m*_S_+1, *m*_I_> parallel transitions at a probing frequency of 7.7 GHz. Labels help in the identification of the transitions. (**c**) Normalized frequency shift δν/ν_0_ dependence as a function of the magnetic field. (**d**) Plot of the normalized transmission as a function of the magnetic field. (**e**) Easy Spin simulation of the CW-ESR powder spectrum at 7.66 GHz based on the parameters given in^25^. Black dashed lines help in recognizing the transitions.

The Vanadyl Phthalocyanine (VOPc for short) molecule is sketched in the inset of Fig. 1a. It presents a Vanadium(IV) ion at the center of a Phthalocyanine ring, which forms a short double bond with an oxygen atom, resulting in an oxovanadium(IV) VO^2+^ group (Vanadyl). This double bond leads to a non-degenerate spin doublet arising from the singly occupied d_xy_ orbital^25^ which is well separated from the higher energy orbitals^25^ and which has negligible orbital contribution, giving a S = 1/2 state. The most abundant (99.75%) isotope of vanadium, ^51^V, has an *I* = 7/2 nuclear spin and gives a multiplet of |*m*_S_, *m*_I_> states^19–21,25^. The calculated^43^ Zeeman energy diagram for a single molecule with the static magnetic field applied along the V = O bond is shown in Fig. 1b. Eight main {∆*m*_S_ = 1, ∆*m*_I_ = 0} transitions constitute the spectroscopic signature of the vanadyl group (blue arrows of Fig. 1b). Full magnetic characterization of solid dispersions with different concentrations of VOPc in its equivalent isostructural diamagnetic analogue, TiOPc, is indeed reported in^25^, together with the characterization of their respective *T*_1_ and *T*_2_ relaxation times. The ESR spectrum of VOPc:TiOPc solid dispersion is dominated by hyperfine splitting features of the Vanadium and can be simulated by using the spin Hamiltonian reported in the Supplementary Information (equation S1), using the following parameters^25^: *g*_*x,y*_ = 1.989, *g*_*z*_ = 1.967, ^*V*^ *A*_x,y_ = 171 MHz and ^*V*^ *A*_z_ = 474 MHz.

Here we investigate four samples with 10% concentration of VOPc in TiOPc (hereafter, samples #1,#2,#3,#4) and one with 30% concentration (sample #5).

### Magnetic resonance in the weak coupling regime

The VOPc:TiOPc samples are placed on the top of the resonator, as shown in Fig. 1a. The resonator is cooled down to cryogenic temperature, and the static magnetic field (*B*_0_) is applied along the axis of the resonator (Fig. 1a). CW transmission spectroscopy is performed by injecting and collecting the MW into the resonator with two antennas (see Methods). Figure 1c and d report the results obtained with sample #4 at different temperatures between 2 and 30 K. The normalized shift of the resonant frequency with respect to zero field, δν/ν_0_ = [ν(*B*_0_) − ν_0_]/ν_0_, and the magnitude of the transmission scattering parameter (*S*_21_) at the resonant frequency normalized to the zero field value, ${\tilde{S}}_{21}={|{S}_{21}({\nu }_{0})|}_{{B}_{0}}/{|{S}_{21}({\nu }_{0})|}_{0},$ are reported as a function of *B*_0_. The measured frequency shifts and transmission signals show resonance peaks that are fully consistent with the hyperfine-split CW-ESR spectra of VOPc^25^. Easy-Spin simulations^43^ of the ESR powder spectrum (Fig. 1e) obtained with the Landé *g-*tensor and the hyperfine tensor values given in^25^ allow us to associate the lines to the corresponding transitions within the |*m*_S_, *m*_I_> multiplet occurring for molecules having the magnetic field applied either parallel or perpendicular to the V = O group.

In particular, for the central portion of the spectrum, intense perpendicular type transition are observed: the line at ≈0.2500 T corresponds to the |−1/2,−7/2> → |1/2,−7/2> transition, the one at ≈0.2950 T corresponds to the |−1/2,7/2> → |1/2,7/2> transition, while the most intense central peak comes from the |−1/2,1/2> → |1/2,1/2> transition, which is almost independent on magnetic field orientation^25^.

**Figure 2:**
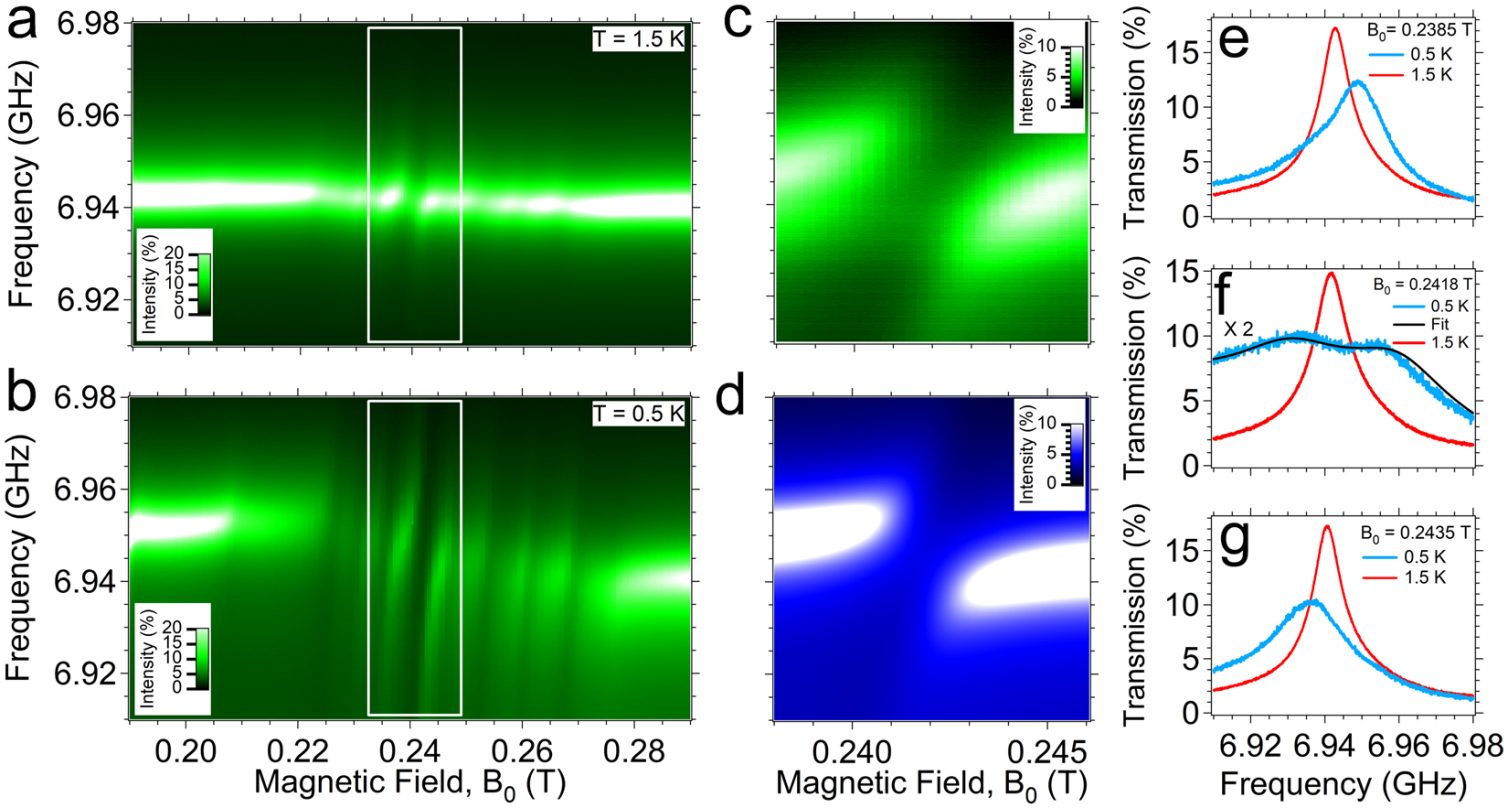
Evidence of Rabi splitting at low temperature. (**a**) Transmission spectral map measured for sample #1 at 1.5 K and −77 dBm. The white rectangle indicates the strongest transition around 0.24 T, which shows a dispersive shift. (**b**) Same spectral map recorded for sample #1 at 0.5 K and −57 dBm. The resonant frequency at zero magnetic field is 6.95 GHz (Res #1). Two anticrossing branches are clearly visible in correspondence of the main transition. (**c**) Detail of the spectral map measured in the range evidenced by the white rectangle in panel (**b**). **(d)** Input-output simulation of the transmission spectral map obtained by equation (1). (**e**–**g**) Sequence of transmission spectra taken at different magnetic fields at 0.5 K (blue line) and at 1.5 K (red line). In particular, (**e**) and (**g**) are taken respectively below and above the resonance field, while (**f**) are recorded at resonance. Rabi splitting is visible at 0.5 K in (**f**). Black lines is the best fit curves based on eq. (1).

Figure 1c and d also display the temperature dependence of δν/ν_0_ and ${\tilde{S}}_{21}$. As the temperature is decreased, the signals become more intense and the frequency shift becomes larger. This behaviour can be related to the increase of the spin polarization, as already observed for other paramagnetic spin systems^31^. Similar results are obtained also for the other samples (Supplementary Information and Figures S7, S8). Figure 2a shows the transmission map measured for sample #1 at 1.5 K and −77 dBm. A main absorption dip in the intensity is visible at *B*_0_ ≈ 0.24 T (white rectangle), with additional weaker absorption dips. The intensity pattern is consistent with the one of the VO^2+^ group (Fig. 1e) and the line shape corresponds to what is expected from CW transmission spectroscopy in the weak coupling regime^31,44^.

### Rabi splitting in the high cooperativity regime

By cooling the sample down to 0.5 K the multiple resonances due to the *I* = 7/2 hyperfine splitting become more intense (Fig. 2b, here for −57 dBm). The central and strongest resonance (area enclosed in the white rectangle on Fig. 2b) clearly shows an avoided crossing at *B*_0_ ≈ 0.2420 T with the splitting of the dispersion in two branches (Fig. 2c). The sequence reported in Fig. 2e,f,g clearly displays the evolution of the transmission spectrum when approaching the resonance field.

In particular, the line shape changes from a single peak for fields lower than the resonance one (panel e), to two distinct peaks on resonance (panel f), and back again to a single peak at higher fields (panel g). We note that such an evolution is substantially different from what is observed in the weak coupling regime (Fig. 1 and 2a and 1.5K data of Fig. 2e,f and g), where only a single peak is visible at resonance. The presence of two anti-crossed branches is the experimental fingerprint of the high cooperativity regime^10,11,13,39,40^. Reproducibility of this behaviour was tested on a different sample and found in similar experimental conditions (Figure S9).

## Discussion

Starting from the Tavis-Cummings model, the transmission spectral map can be simulated using the input-output formalism:

1 $${S}_{21}(\nu )=\frac{{\kappa }_{ext}}{i({\nu }_{0}-\nu )+{\kappa }_{ext}+\frac{{\kappa }_{int}}{2}+\frac{{\Omega }^{2}}{i({\nu }_{S}-\nu )+\gamma }}$$

In this case, the second quantization and the scattering matrix method are combined to derive the complex transmission spectra of the whole system (resonator coupled to the spin ensemble)^37,40^. A Lorentzian distribution for the spin linewidth is assumed^45^ and checked to best fit the experimental spectra (see Supplementary Information for further discussion on this point). In equation (1), κ_*ext*_ and κ_*ιnt*_ are the -so called- external and internal decay rate of the resonator respectively, and they were fixed at κ_*ext*_ = 2.1∙10^4^ Hz and κ_*ιnt*_ = 1∙10^6^ Hz from fittings of the zero-field transmission signal preliminarily determined (see also Figure S4 and Table S1), while γ is the Half Width at Half Maximum (HWHM) spin linewidth. Although equation (1) well reproduces the transmission spectra in the whole temperature range (black lines in Fig. 2e and g), for sake of simplicity we use a simpler lumped element model to fit the spectra in the weak coupling regime (equation S4 and Figure S11). Since high cooperativity is observed only for the most intense line, hereafter we focus our attention on this transition.

**Figure 3:**
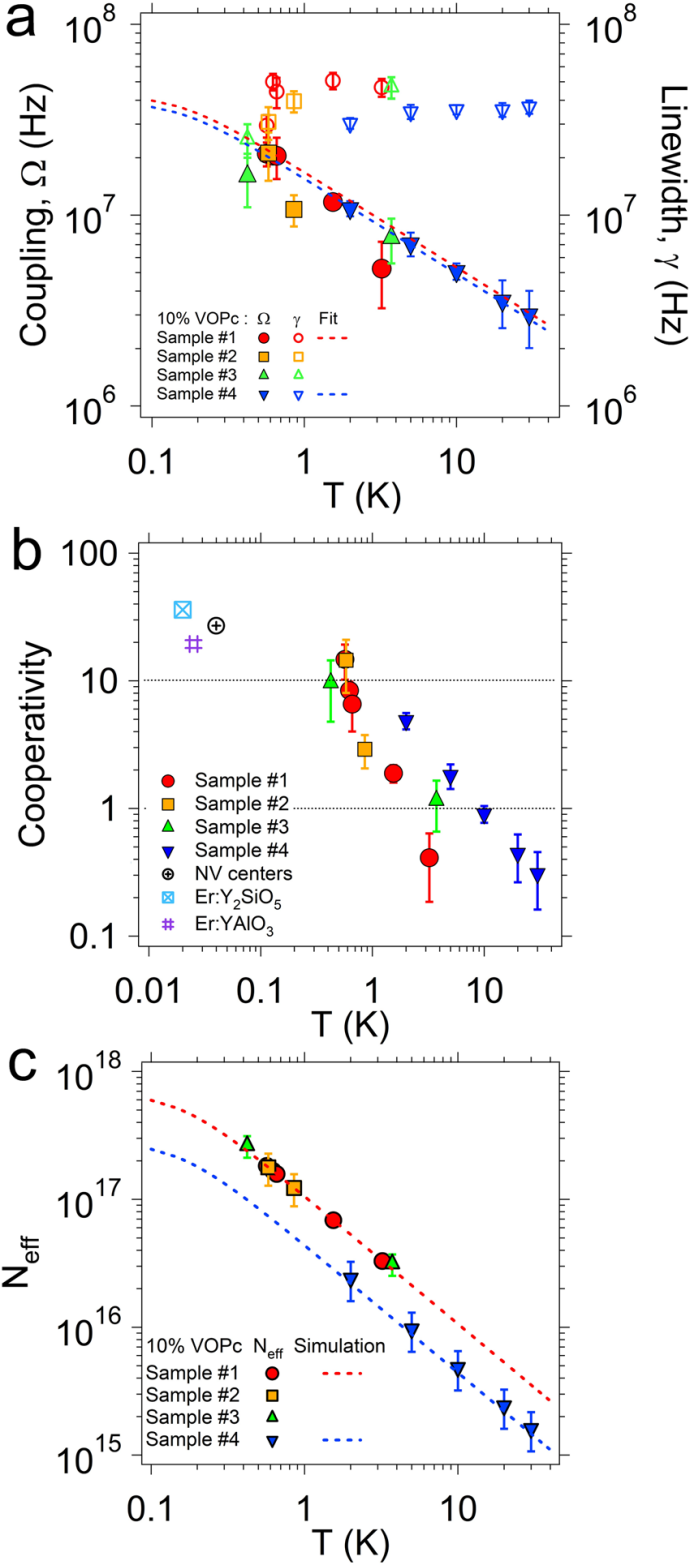
Coupling regimes. (**a**) Coupling rate (Ω) and spin ensemble linewidth (γ) as a function of the temperature for the main line of all the 10% VOPc:TiOPc samples. Dashed lines are fits based on equation (2). (**b**) Cooperativity as a function of the temperature calculated from the curve fitting results reported in (**a**). Values from literature are added for comparison: the NV centers point is taken from^10^, while the Er^3+^ ions are taken from^13^ and^14^. (**c**) Effective number of spin extracted from Ω according to equation (2) as a function of the temperature for all the samples. For sample #1 and #4, dashed line indicates the values given by simulations (Supplementary Information).

In Fig. 3a we plot the temperature dependence of the Ω and γ values obtained from the data fit of the data of all the samples with the 10% VOPc:TiOPc concentration. Over the 1–30 K temperature range, the linewidth of the main line is γ ≈ 35 ± 4 MHz, with essentially no temperature dependence. This value is well reproducible over the different specimens we have measured (different symbols in Fig. 3a).

In the case of homogeneous broadening only, the CW-ESR linewidth for the 10% VOPc:TiOPc samples, estimated from *T*_1_ and *T*_2_ (or *T*_m_) values provided by pulsed EPR experiments (Figure S2 and Equation S2), is *γ*_*hom*_ ~ 5 MHz at 300 K (and 0.5 MHz < *γ*_*hom*_ < 3 MHz at 5 K, see Supplementary Information). This leads us to conclude that the main contribution to the line broadening, in our experiments, comes from inhomogeneous broadening and that our linewidth is not simply limited by single molecule *T*_1_ and *T*_2_ relaxation times. This also suggests that the fitted γ value constitutes an upper bound for the spin linewidth that reflects all our experimental conditions rather than intrinsic molecular features. A detailed discussion about the inhomogeneous broadening is given in the Supplementary Information.

The coupling rate Ω of the main transition monotonically increases as the temperature decreases, as expected from thermal variation of the spin polarization. This is also confirmed by the enhancement of the frequency shifts and of the intensity of the transitions (Figs 1c,d and 2a,b,e,g). Due to the stability in γ, the cooperativity is dominated by the quadratic dependence of Ω and, hence, it increases of about one order of magnitude as the temperature decreases from 30 K to 0.5 K (Fig. 3b).

The highest coupling value is obtained for sample #1 at 0.5 K and it corresponds to the data shown in Fig. 2b–g. The best fit for this sample (Fig. 2d and f) gives Ω = 21 ± 3 MHz and γ = 30 ± 4 MHz, resulting in a cooperativity value *C*≈15 at 0.5 K. This corroborates the fact that our system is in the high cooperativity regime (*C* > 1) and coherent spin-photon coupling is taking place. It is worth to note that, due to the overestimation of γ discussed above, this value can be considered as a conservative estimation for the cooperativity. The anti-crossing and Rabi Splitting of the transmission spectra was found also in sample #3, giving Ω = 16 ± 5 MHz, γ = 25 ± 6 MHz and *C* ≈ 10 (Figure S9).

Additional cooperativity values reported in literature for NV centers^10^ and Er^3+^ ions in inorganic crystals^13,14^ are included in Fig. 3b for comparison. This is limited to few examples of ensembles of isolated spins (NV centers and Er^3+^ impurities in inorganic hosts) magnetically coupled to microwave photons in planar superconducting resonators since cooperativity depends on different parameters that change from one experiment to another. Notably, the cooperativity found in our experiments at 0.5 K is comparable to values obtained with conventional superconductors and spin impurities in inorganic matrix at much lower temperatures.

We now provide more quantitative analysis about the number of spins that are electromagnetically coupled to the resonators. According to the Tavis-Cummings model, the collective coupling strength related to the j^th^ transition is^3,40^

2 $${{\rm{\Omega }}}_{j}={{\rm{\Omega }}}_{S,j}\sqrt{{N}_{eff,j}}={{\rm{\Omega }}}_{S,j}\sqrt{{N}_{0}{p}_{j}(T)},$$

where Ω_S,j_ is the single spin coupling strength and the *N*_*eff*_ is effective number of spins coupled to the photons. The latter is *N*_*eff*,j_ = *N*_0_ *p*_j_ (*T*), being *N*_0_ the total number of spins located within the volume of the resonator mode, and *p*_j_ *(T)* the temperature dependent polarization factor accounting for the thermal population ratio. For the VOPc molecule, *p*_*j*_(*T*) can be calculated from the Brillouin function with  *J* = 1/2^25,31^. We first estimate *N*_0_ (${N}_{0,sim}$ in Table 1 and in the following) from the volume of the spatial distribution of the resonant mode calculated by means of the full 3D electromagnetic simulations (Supplementary Information). Then, the *Ω*_j_-vs-*T* dependence is fitted with equation (2) (dashed lines of Fig. 3a and Figure S13), by keeping the product ${{\rm{\Omega }}}_{S,j}\sqrt{{N}_{0}}$ as a fitting parameter. The single spin coupling strength is estimated as ${{\rm{\Omega }}}_{S,j}=({{\rm{\Omega }}}_{S,j}\sqrt{{N}_{0}})/\sqrt{{N}_{0,sim}}$ (${{\rm{\Omega }}}_{S,fit}$ in Table 1). Finally, from the knowledge of *p*_j_ *(T)* and of the fitted ${{\rm{\Omega }}}_{S,j}$, the effective number of spins (*N*_*eff*,j_) is calculated as a function of the temperature, giving the dashed lines of Fig. 3c. The resulting *N*_0_ and *Ω*_*S*_ for the main transition for all the 10% VOPc:TiOPc samples are reported in Table 1. The small differences observed for different samples can be ascribed to the different mode volume of the resonators that we have used (Supplementary Information). In Table 1 the mean cavity photon number (*n*_p_) estimated for each experiment according to^39^ is also reported. Since different incident powers were used during the experiments, the values reported in Table 1 correspond to the maximum powers and provide the upper limits for *n*_p_. The comparison with the effective number of spins shows that the condition *n*_p_ ≪ *N*_eff_ always holds in our experiments^9^. The fitted *Ω*_*S*_ are consistent with the values obtained from the independent estimation of the transition matrix elements of the main transition (see Supplementary Information).

**Table 1:** Spin number and mean cavity photon number for the main transition of VOPc.

Sample	*N*_0,*sim*_ (spin)	Ω_*S*,*fit*_ (Hz)	*n*_*p*_
#1	(6.4 ± 0.9) ∙ 10^17^	0.05 ± 0.01	≤6.4 ∙ 10^9^
#2	(6.4 ± 1.1) ∙ 10^17^	0.04 ± 0.01	≤4.8 ∙ 10^9^
#3	(7.1 ± 1.1) ∙ 10^17^	0.04 ± 0.01	≤1.6 ∙ 10^9^
#4	(2.6 ± 0.8) ∙ 10^17^	0.07 ± 0.01	≤8.3 ∙ 10^11^

The maximum number of spins coupled to the resonator (*N*_0,sim_) and the values of single spin coupling (Ω_S_) obtained respectively from the electromagnetic simulations and from the fitting of *Ω*-vs-*T*  dependence (Supplementary Information) are reported. The last column reports the mean cavity photon number for the maximum input power used for each sample.

## Conclusions

In summary, we have shown that, by a suitable choice of the molecule and the experimental parameters, it is possible to tune the magnetic coupling between molecular spin ensembles and microwave photons in a superconducting planar resonator and to drive the system in a highly cooperative regime. Rabi splitting in the energy spectrum is clearly observed and data analysis allows us to extract the collective coupling rate and the spin linewidth, giving high cooperativity $C\approx 15$ at 0.5 K. This *C* value compares well to those measured on the NV centers and the Erbium impurities with conventional superconducting circuits^2,10,11,13,14^. The temperature at which high cooperativity is found in our experiments is one order of magnitude higher than the typical working temperatures of circuit-QED experiments (≈10–50 mK)^10,11,13^. This is also partially due to the higher critical temperature of YBCO with respect to conventional Aluminum and Niobium superconductors. Our analysis reveals that the spin linewidth is limited by the inhomogeneous broadening and not simply by the *T*_1_ and *T*_2_ relaxation rates of the molecule, suggesting that further improvements are possible. It is worth noting that the extrapolation of the Ω values of 10% VOPc:TiOPc samples in the mK region (dashed lines of Fig. 3a) shows that also Ω could be further enhanced. Finally, we point out that, for *T* < 0.1 K, the hyperfine energy levels are no longer equally populated and this is expected to provide additional enhancement of the collective coupling^46,47^. This suggests that molecular spin ensembles can fully enter in the so-called strong coupling regime (for which Ω ≫ γ) and that, at low temperatures (≈20–50 mK), comparable cooperativity values with respect to NV centers and Er^3+^ ions can be reached (dashed lines of Fig. 3a).

These results open the possibility to integrate molecular spin centers with long coherence times in hybrid architectures based on superconducting quantum circuits. The discussion of the key parameters in our experiments indicates that there are margins for improving cooperativity. Moreover, the possibility to sublimate VOPc under vacuum and the possibility to control its deposition on different surfaces, make this molecule interesting in view of the fabrication of hybrid devices.

## Experimental Methods

### Samples

5%, 10% and 30% VOPc:TiOPc solid dispersions were prepared following the same procedure reported in^25^ and compressed in polycrystalline pellets. Structural phase homogeneity has been checked for all samples through Powder X-ray Diffraction analysis (Figure S1). X-ray fluorescence (Supplementary Information) analyses were used to estimate the effective doping percentages for the Vanadium, giving concentration values of 5.0 ± 0.5%, 10 ± 1%, and 29 ± 1% respectively.

### Experimental Set-up

Transmission spectroscopy experiments are carried out by exploiting the dipolar coupling between the collective magnetic moment of the spin ensemble and the magnetic component of the fundamental mode of a YBCO/Sapphire superconducting coplanar resonator^39^ (Fig. 1a, Fig. S3 and Supplementary Information). Two resonators with different sizes, Res #1 (for Sample #4) and Res #2 (with Samples #1,#2,#3,#5) are used. The dimensions of the YBCO resonator have been optimized in order to maximize the number of spins involved (Supplementary Information). The *Q* factors for the bare resonators at *T* ≤ 2 K are of the order of ≈6∙10^3^ for Res #1 and ≈10^4^ for Res #2, and correspond to a decay rate $k\approx \,1{\rm{MHz}}$ (Table S1 and Supplementary Information). In our set-up, the transmission scattering parameter of the resonator^48^ is measured as a function of frequency by means of a Vector Network Analyzer (VNA) for different applied *B*_0_, as previously reported in^31,39,40^. The value of the incident microwave power, which is estimated at the launching antenna by taking into account the losses of the MW line and the attenuators (Supplementary Information), ranges between −77 and −45 dBm. Preliminary checks are performed to choose input powers low enough to prevent line saturation effects^49^. Further details of our experimental set-ups are given in the Supplementary Information.

## Electronic supplementary material

 Supplementary Information
