# Supplementary material for: Coherent coupling between Vanadyl Phthalocyanine spin ensemble and microwave photons: towards integration of molecular spin qubits into quantum circuits
Source: Sci Rep. 2017 Oct 12;7:13096. doi: 10.1038/s41598-017-13271-w (PMC5638858; doi:10.1038/s41598-017-13271-w)
Supplement: Supplementary file 1 — Supplementary Information [file 41598_2017_13271_MOESM1_ESM.pdf]

SUPPLEMENTARY MATERIAL TO:

Coherent coupling between Vanadyl Phthalocyanine spin ensemble and microwave photons: towards integration of molecular spin qubits into quantum circuits

C. Bonizzoni*[a,b], A. Ghirri[b], M. Atzori[c], L. Sorace[c], R. Sessoli[c], and M. Affronte[a,b]

[a]Dipartimento di Scienze Fisiche, Informatiche e Matematiche, Università di Modena e Reggio Emilia, via G. Campi 213/A 41125 Modena, Italy

[b]Istituto Nanoscienze – CNR, via G. Campi 213/A 41125 Modena, Italy

[c] Dipartimento di Chimica "Ugo Schiff" & INSTM RU, Universit. degli Studi di Firenze, Via della Lastruccia 3, 50019 Sesto Fiorentino (Firenze), Italy

*Correspondence to Claudio Bonizzoni (CB): claudio.bonizzoni@unimore.it

ADDITIONAL INFORMATION ON SAMPLES.

Samples preparation. Crystalline dispersions of VOPc in the diamagnetic analogue TiOPc (Phase II polymorph) were prepared according to a previously reported procedure [1], by adjusting the stoichiometric ratio of the VOPc and the TiOPc starting materials (Sigma-Aldrich, Phase IV polymorphs) to obtain nominal concentrations of 5%, 10% and 30% of VOPc in TiOPc. X-ray fluorescence (XRF) analyses were used to estimate the effective doping percentage by comparing the intensity of the XRF intensity of vanadium K_{β} emission of the prepared compounds to that of a calibration curve obtained by mixing weighted amounts of the pure compounds (VOPc and TiOPc) in the 1-50% concentration range. The effective concentration of vanadium is 5.0±0.5%, 10±1%, and 29±1%, respectively. Powder X-ray Diffraction (PXRD) patterns were recorded to check the structural phase homogeneity of all samples polymorph (Figure S1).

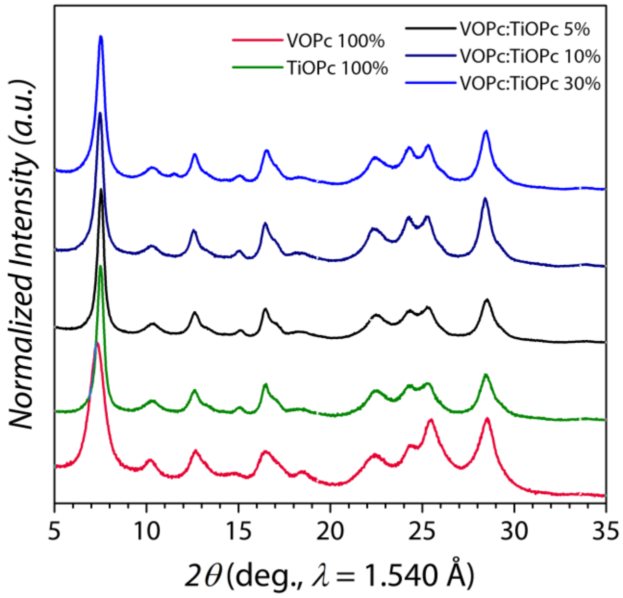

Figure S1 Experimental PXRD patterns (5-35°, 2θ) for VOPc:TiOPc crystalline dispersions (Phase-II) at different VOPc concentrations (see legend). PXRD pattern of pure VOPc-II and pure TiOPc-II are also reported for comparison.

X-ray Fluorescence. X-ray fluorescence analyses were performed with a *WD-XRF Rigaku PrimusII* spectrophotometer.

Powder X-ray Crystallography. Wide-Angle Powder X-ray Diffraction (PXRD) patterns on polycrystalline samples were recorded on a *Bruker New D8 Advance DAVINCI* diffractometer in a theta-theta configuration equipped with a linear detector. The scans were collected within the range 5-35° (2θ) using CuK_α radiation (λ = 1.540 Å).

Standard ESR characterization.

The standard ESR characterization of VOPc in TiOPc at different doping levels has been previously reported in [1]. The Spin Hamiltonian used to analyze the properties of VOPc molecule is reported in equation (S1) for clarity.

$$H = \mu_B \hat{S} \cdot g \cdot B_0 + \hat{I}_V \cdot {}^{V}A \cdot \hat{S} + {}^{N}A \sum_{i=1}^{4} \hat{I}_{N_i} \cdot \hat{S}$$

(S1)

In equation (S1), g is the Landè g-tensor, ^{V}A the hyperfine tensor describing the interaction between the spin $\hat{S}$ of the VO^{2+} group with the nuclear spin of the Vanadium ($\hat{I}_V$) and ^{N}A the scalar describing the interaction between the spin $\hat{S}$ and the nuclear spin of nitrogen, for

which the anisotropy is not resolved. For the 10% diluted molecule, the hyperfine interaction with N is completely unresolved, and the best simulation of the EPR spectrum was obtained by using the following parameters: $g_{x,y}$=1.989(1), g_z=1.967(1), $A_{x,y}$=0.0057(1) cm^{-1}, A_z=0.0158(1) cm^{-1} including a Gaussian H-strain to account for the unresolved hyperfine coupling [2].

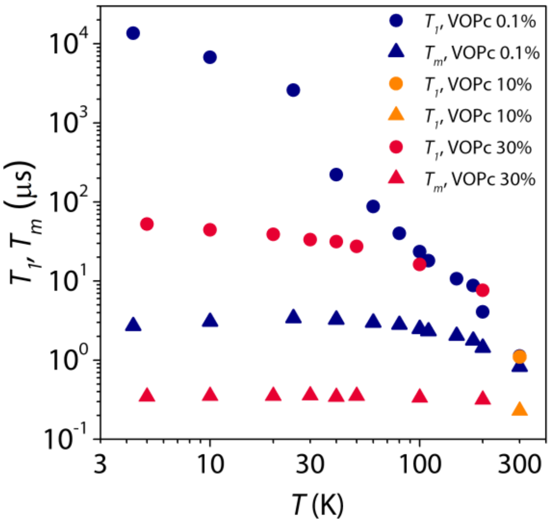

Figure S2 Temperature dependence of the relaxation times T_1 and T_m for VOPc:TiOPc solid dispersions with different concentrations: 30% (4-200 K), 10 % (300 K) and 0.1 % (5-300 K). Data for 0.1% and 10% samples were already reported in [1].

Figure S2 reports the temperature dependence of the relaxation time T_1 and of the memory time T_m (T_m being an upper bound of T_2) for the 30% sample and the 0.1 % sample (this last one from [1]). It is evident that in both cases T_1 increases significantly when decreasing the temperature while a much less marked increase is observed for T_m; furthermore, while we have $T_1 = 10^4$ µs and $T_m = 2$ µs for the 0.1 % sample at 5 K, the 30 % sample features $T_1 = 52$ µs and $T_m = 0.35$ µs at 4 K. We expect intermediate values for the 10% sample. The observed temperature dependence is consistent with that of other Vanadyl complexes [3].

ADDITIONAL INFORMATION ON YBCO RESONATORS.

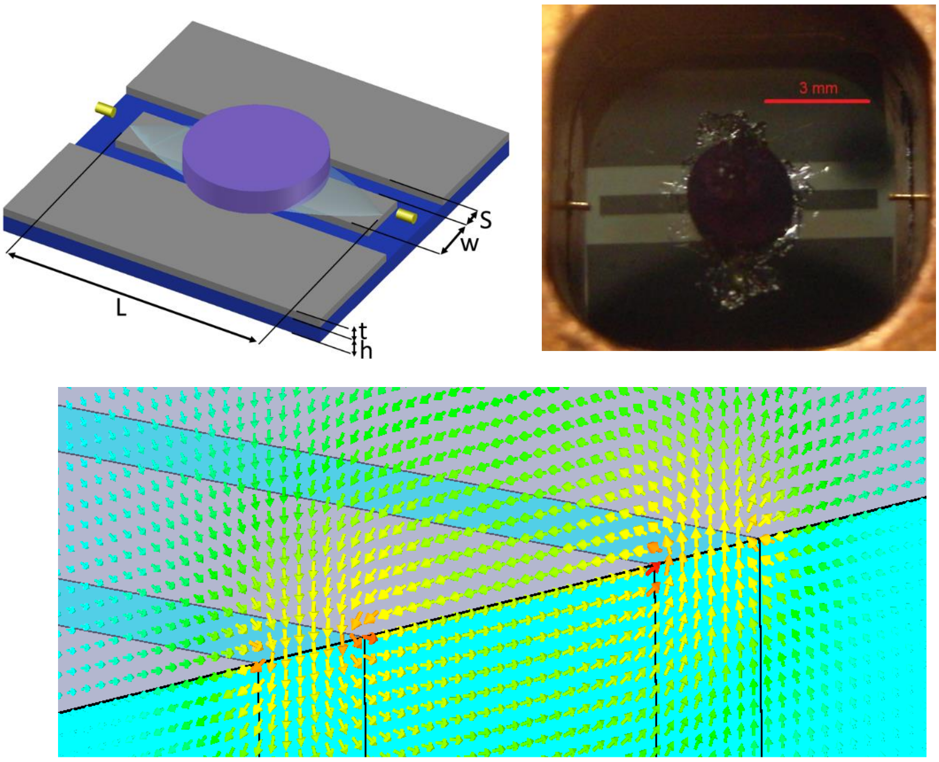

Figure S3 (top left) Sketch of the resonator with a cylindrical sample (purple) positioned in the center. The labels indicate the dimensions reported in the text. (top right) Photo of Res #1 with sample #1 loaded on it. (bottom) Electromagnetic simulation of the spatial distribution of B_1 on a plane perpendicular to the axis of Res #2 (taken at half of the length of the resonator). The light blue region represents the Sapphire substrate, while the grey regions correspond to the resonator and to its lateral ground planes. The arrows represent the direction of the magnetic field, and their colour scale (arbitrary units) represents the intensity of B_1.

Half-wavelength coplanar resonators support the transmission of a fundamental Quasi-Transverse Electro-Magnetic (QTEM) mode. The magnetic lines of force of the MW field wrap around the resonating strip and lie almost entirely on a plane perpendicular to the longitudinal axis of the strip (Figure S3 bottom). The magnetic component of the MW field has its maximum in the middle of the conducting strip [4], where the magnetic sample is usually positioned (Figure S3 and Figure 1.a of the main text). With the static magnetic field parallel to the axis of the resonator, this geometry allows the detection of perpendicular type of magnetic transitions. The Sapphire substrate has a thickness h = 430 μm, while the

thickness of the YBCO film is t = 330 nm (Figure S3). We used two resonators: Res#1 has W = 600 μm, S = 800 μm, L = 8 mm, while Res#2 has W = 200 μm, S = 73 μm and L = 8 mm. For both the bare (empty) devices, the transmission spectrum has a Lorentzian line shape (Figure S4). The small asymmetry in the Lorentzian tails is due to the electromagnetic coupling to the local environment given by the antennas, the lateral ground planes and the shielding box [5]. We fit the transmission spectra of the bare resonator with equation (1) of the main article, in which we force Ω and γ to be zero (i.e. no coupling to the sample). Best fit parameters for the spectra of Figure S4 are reported in Table S1. The quality factor, Q, is then calculated from the fitted parameters according to $Q = \nu_0/(k_{int} + k_{ext}/2)$. The consistency of the fitted Q values has been checked by independently fitting the transmission spectra of Figure S4 with the curve reported in [4] and by a direct measure of the Q factor on the VNA screen according to the "-3 dB rule" [6].

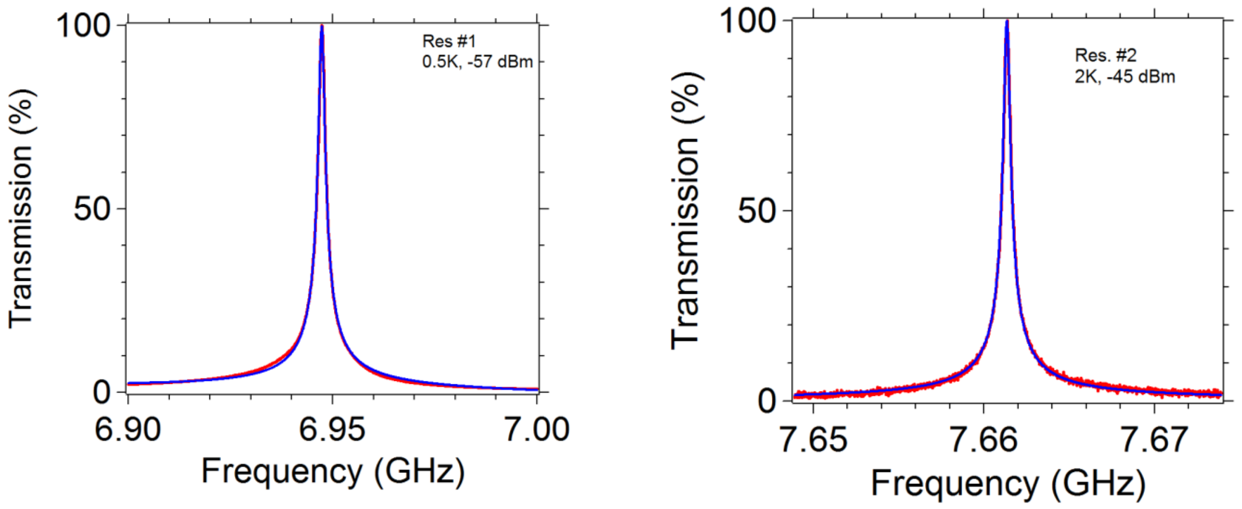

Figure S4 Transmission spectra for Res #1 (left) and #2 (right). The corresponding temperatures and input powers are indicated in the legends. Blue lines are fits with the curve described by equation (1) of the main article.

Res	k_{ext} (Hz)	k_{int} (Hz)	ν_0 (Hz)	Q-factor
#1	$2 \cdot 10^4$ to $3.5 \cdot 10^5$	$8.9 \cdot 10^5$ to $1 \cdot 10^6$	$6.947 \cdot 10^9$	6476±910
#2	$1.35 \cdot 10^5$	$7.28 \cdot 10^5$	$7.661 \cdot 10^9$	9630±1300

Table S1. Best fit parameters for the zero field transmission spectra of Figure S4 obtained by means of equation (1) of the main paper. The Q-factor calculated from k_{ext}, k_{int} and ν_0 is added for clarity. The temperature is 0.5 K for Res #1 and 2K for Res #2.

The Q factors are of the order of $\approx 6{\cdot}10^3$ for Res#1 and $\approx 10^4$ for Res #2, and correspond to a total decay rate $k = k_{int} + k_{ext}/2$ of $\approx$ 1MHz. The Q-factors and the resonant frequencies are stable over the entire studied temperature range, also below 2 K (Figure S5) [4].

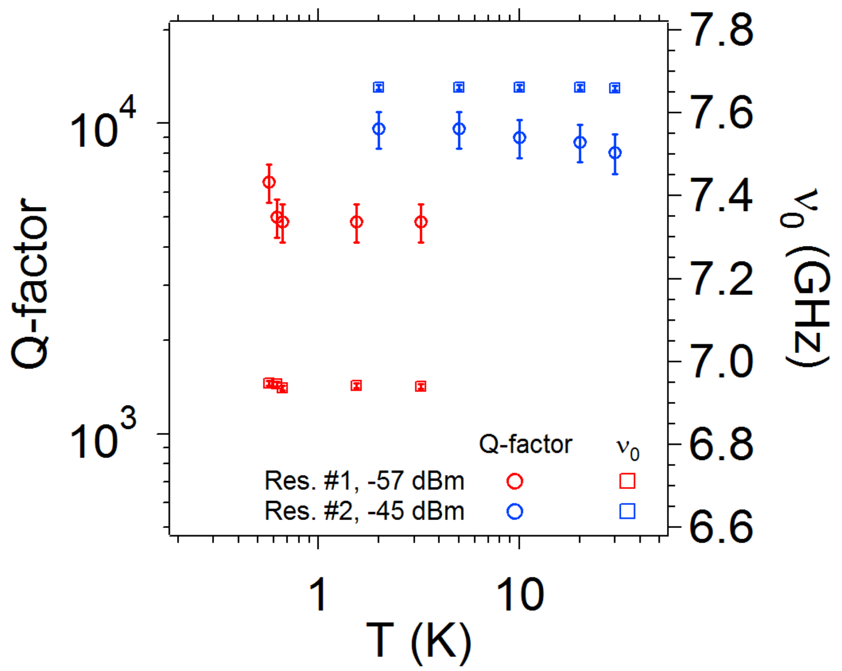

Figure S5 Characterization of the bare resonators in zero field Q-factor and of the resonant frequencies for Res#1 and #2 as a function of the temperature. The input powers are reported in the legend.

The value of k_{ext} strongly depends on the position of the micro-antennas with respect to the resonant strip and can change of one order of magnitude from one experiment to another. Table S1 reports a typical range for the fitted k_{ext} values. In this range the resonator is

undercoupled from the feedlines, thus the exact position of the micro-antennas only slightly affects the value of the Q factor.

When the resonator is loaded with a sample pellet we observe a decrease of the intensity of the zero-field transmission and a corresponding increase of its width. The final effect is a slight increase of the k_{int} parameter up to ≈1 MHz.

In a preliminary investigation, the size of the resonator was changed in order to optimize the performances. The width of the resonator Res#1 was finally fixed at 600 μm in order to increase the volume of the resonant mode and, hence, to increase the number of spins involved in the coupling (electromagnetic simulation section and dashed lines of Figure 3.c of the main paper). Wider resonators also give flatter gradients in the oscillating magnetic field and help in the reduction of its inhomogeneity.

ADDITIONAL INFORMATION ON SET-UP.

Sample #4 was used for preliminary measurements with Res#2 by means of the set-up described in [7], which is installed in a Quantum Design Physical Properties Measurement System (PPMS) working in the temperature range between 2 and 300 K. Samples #1, #2, #3 and #5, were measured with Res#1 by means of an Oxford Heliox VL ^{3}He refrigerator (base temperature of 300 mK) and Oxford triaxial superconducting solenoid (0-9 T on the z-axis).

Figure S6 shows a simplified scheme of the set-up installed on the Oxford Heliox VL ^{3}He cryostat. In the input line, two attenuators, one at room temperature (30 dBm) and one at the 1.5 K thermalization stage (20 dBm) are used to decrease the input power and the heat load on the sample. In the output line, a room temperature amplifier (38 dB) is used for the amplification of the signal. An Agilent PNA 26.5 GHz Vector Network Analyzer (VNA) is used for the generation of the probing signal and for the acquisition of the transmission spectra. The attenuation of the coaxial line (17 dB) is estimated from preliminary room temperature calibrations and has been taken into account for the calculation of the input power on the antennas and of the mean photon number.

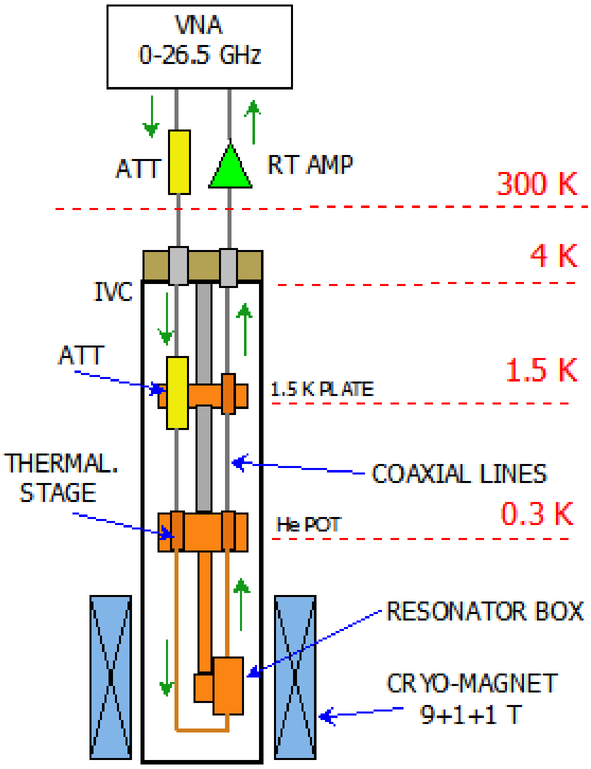

Figure S6 Sketch of the experimental set-up used for the measurements down to 300 mK. Only the Inner Vacuum Chamber (IVC) is shown for clarity.

ADDITIONAL SPECTROSCOPIC RESULTS.

Figure S7 shows the transmission spectra for the 30% (Sample #5). The hyperfine structure is the expected one for a S=1/2, I=7/2 powder. Hyperfine signal has wider transitions with respect to sample #1. This is an effect of the higher (3 times) spin concentration, which increases the dipolar interaction among the spins. Additional measurements performed on a 5% sample (not shown) revealed that, despite the higher dilution, there is no significant reduction of linewidth with respect to the 10% sample. The pellets obtained by compressing the 10% solid dispersion give consistent and reproducible spectra (Figure S8).

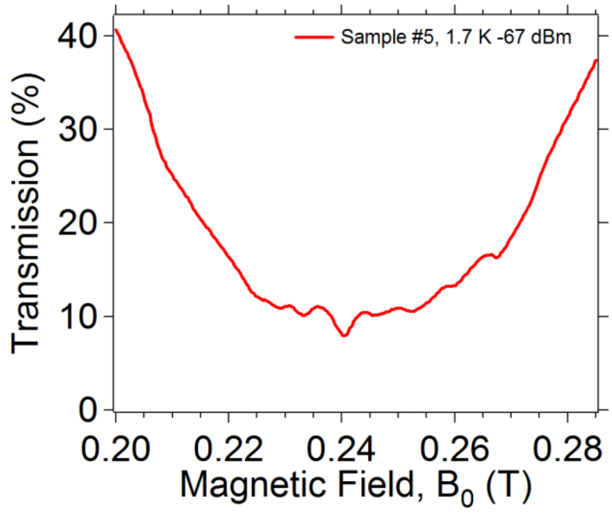

Figure S7 Transmission signal measured for the sample #5 with concentration 30%.

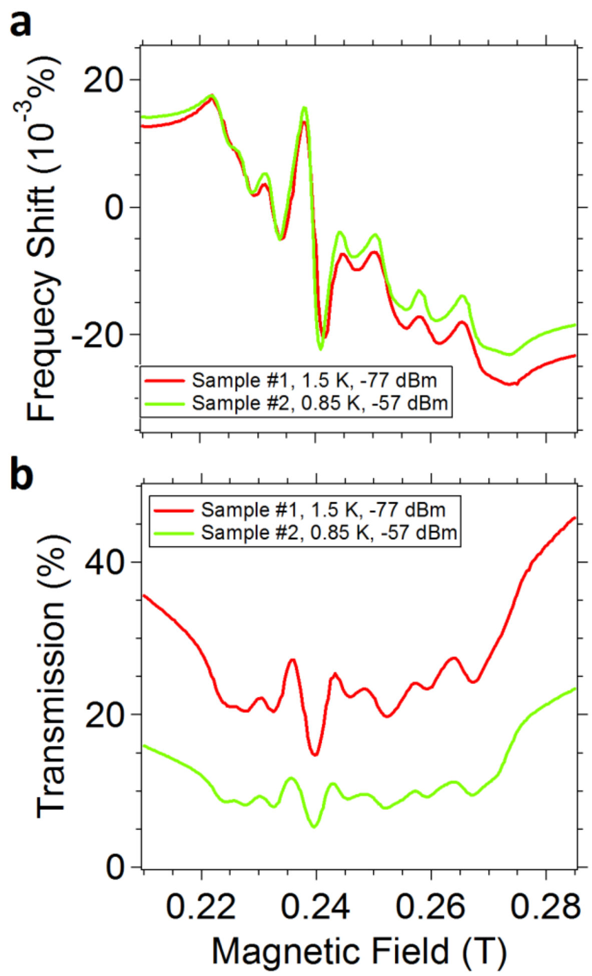

Figure S8 Reproducibility of 10% samples. Frequency shift (a) and transmission (b) of sample #1 and #2. The experimental parameters are reported in the legend.

Figure S9 shows the high cooperativity measured in sample #3 at 0.4 K and -52 dBm. The avoided crossing is visible in the transmission map. The transmission spectra changes from one peak to the convolution of two peaks when the resonance is reached (Figure S9.c and S9.d). Moving to higher magnetic fields a single peak in transmission is recovered (Figure S9.e). Curve fittings are performed according to equation (1) of the main article (Figure S9.b and S9.d), and give $\Omega = 16 \pm 5$ MHz, $\gamma = 25 \pm 6$ MHz and a cooperativity $C \approx 9.4$. Since the linewidth is similar to the one of sample #1, the difference is due to the different N_0.

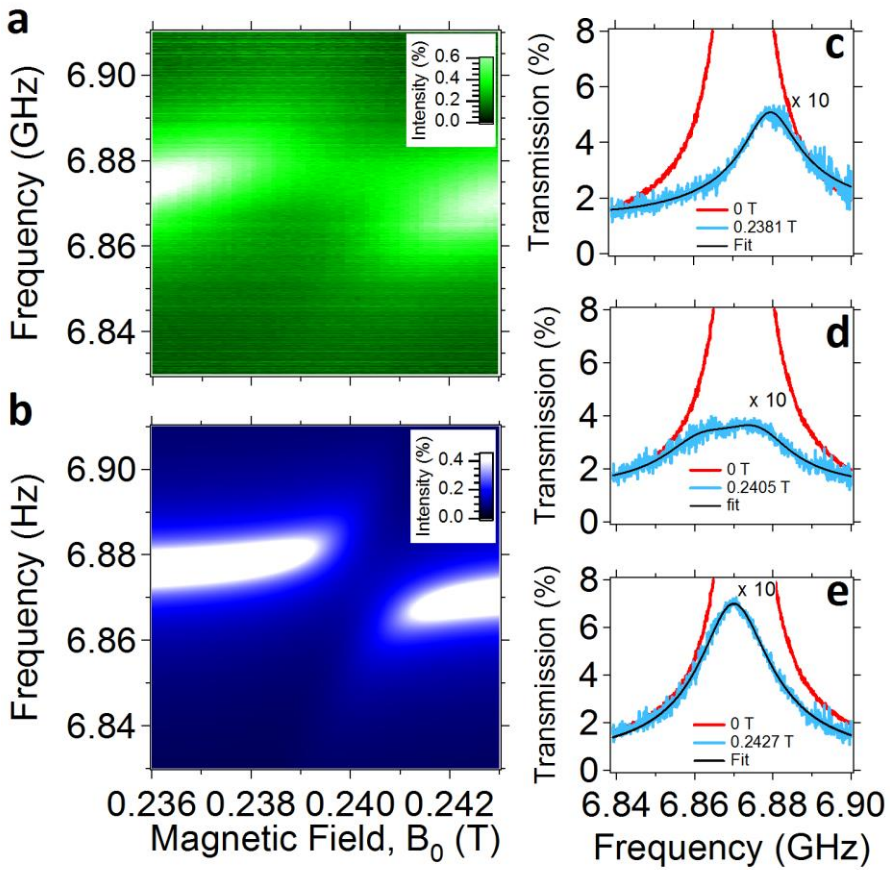

Figure S9 High cooperativity in sample #3. (a) Transmission spectral map measured for sample #3 at 0.4 K and -52 dBm. (b) Simulated transmission spectral map calculated according to equation (1) of the main article. (c) and (e) Transmission spectra, measured for two magnetic fields slightly below and above the resonance field, in which only one peak is visible. (d) Transmission map at resonance. Black lines show the fit curves calculated with equation (1) of the main paper.

SPIN LINEWIDTH AND SOURCES OF LINE BROADENING.

The intrinsic molecular line-broadening limit. In the case of pure homogeneous broadening due to the intrinsic features of the spin centers, the CW-ESR linewidth of an ensemble of magnetic molecules is given by equation (S2) [8]:

$$\gamma_{hom} = \frac{1}{2T_1} + \frac{1}{T_2}\sqrt{1 + \gamma_e^2 B_1^2 T_1 T_2}$$

(S2)

Here, $1/T_1$ and $1/T_2$ are the spin-lattice and the spin-spin relaxation rate respectively, while γ_e is the electronic gyromagnetic ratio and $B_1$is the MW magnetic component. Since the experiments are performed at low input power (and, hence, low intensities of B_1) to prevent the saturation of the magnetic transitions, equation (S2) can be simplified by assuming $\gamma_e^2 B_1^2 T_1 T_2 \ll 1$ (no saturation limit). By substituting the values of T_1 and T_m (assumed equal to T_2) obtained by Pulsed EPR experiments (Figure S2) in equation (S2) one can estimate γ_{hom} at 5 K for the 0.1 % sample and for the 30 % sample. This provides γ_{hom} ~ 0.5 MHz (0.1 %) and γ_{hom} ~ 3 MHz (30 %), with the 10% sample being expected to have an intermediate value between these two. This is also consistent with the value of γ_{hom}~ 5 MHz calculated for the 10% sample at 300 K using the T_1 and T_m values reported in [1]. It is at any rate clear that the estimated γ_{hom} is much lower than the experimentally observed linewidth γ ≈ 30 ± 4 MHz, suggesting that other sources of line broadening are active in our experiments.

Sources of line broadening. The most common one comes from the *magnetic dipolar interaction* among the spins, as evident by the data reported in [1], where dilution of the sample from 10% to 0.1% drastically narrows the linewidths of the CW-ESR spectra. In diluted samples the distances between the spin centers are random and the local field due to the magnetic dipolar interaction differs from one site to the other. Thus, one would expect an inhomogeneous broadening.

A further contribution to line broadening originates from the *anisotropy of the g tensor and of the hyperfine interaction* (see equation S1): the powder form of our solid dispersion contributes to average the electronic transitions over all the possible orientations with respect to the direction of static magnetic field, resulting in inhomogeneously broadened

lines of the spectrum. In addition, the VOPc molecules contain other isotopes with non-zero nuclear spin (^{14}N (I=1), ^{15}N (I=1/2) and ^{1}H), resulting in additional, *unresolved hyperfine interactions* that act as source of inhomogeneous broadening [9].

Other sources of broadening are given by the resonator's geometry. The *dimensions of the samples* (3-5 mm) with respect to the length of the resonator (8 mm) suggest that the amplitude of the oscillating magnetic component B_1 is not uniform along the length of the sample so that the spins are probing different B_1. Secondly, as reported in [10], a *static magnetic field gradient* of ≈0.9 T/m is acting along the longitudinal axis of the device. This implies that the applied external static magnetic field is not uniform over the volume of the sample, giving a linear distribution of different precession frequencies for a given B_0. For instance, a sample with a length of 3 mm can experience a static magnetic field difference between its extremes of ≈30 gauss.

These considerations lead us to conclude that the different mechanisms may contribute to line broadening in our low temperature experiments and we expect an inhomogeneous broadening of the transition lines.

SPIN DISTRIBUTON FOR THE INHOMOGENEOUS LINE BROADENING

As reported in [11], inhomogeneous broadening can be treated by assuming a Lorentzian or a Gaussian functional form for the distribution of the spectral density of the spin ensemble. The analysis of Figure 2d, 2.f and S9 carried out with equation (1) is assuming a Lorentzian distribution, which gives a unique parameter (γ) to describe the spin linewidth and, hence, the broadening effects. To check the robustness of this assumption, the analysis of the data of Figure 2.d was repeated by means of equation (S3), which is derived from the results given by [11]. Here, a Gaussian density with central frequency ν_S and HWHM Δ is assumed to model the inhomogeneous broadening. In this way, the broadening is now described by two independent parameters: the inhomogeneous part (Δ), which is responsible for decoherence, and the homogeneous part, γ_{hom}. The symbol erfc() denotes the complex error function of its argument.

$$S_{21}(\nu) = \frac{\kappa_{ext}}{i(\nu_0 - \nu) + \kappa_{ext} + \frac{\kappa_{int}}{2} - \Omega^2 \frac{\sqrt{\pi \ln 2}}{\Delta} e^{-\left(\frac{(\nu_S - \nu) + i\gamma_{hom}}{\Delta / \sqrt{\ln 2}}\right)^2} erfc\left(-i \frac{(\nu_S - \nu) + i\gamma_{hom}}{\Delta / \sqrt{\ln 2}}\right)}$$

(S3)

Curve fitting with equation (S3) are performed by fixing $\gamma_{hom} = 2$ MHz (see the spin linewidth section), and by keeping Δ and Ω as free parameters. All the other parameters were fixed at the same values used for equation (1) of the main text. The result is shown in Fig. S10 (solid line), while the best fit parameters are reported in Table S2. Best fits obtained for different choices of γ_{hom} are added for comparison (dashed lines of Figure S10).

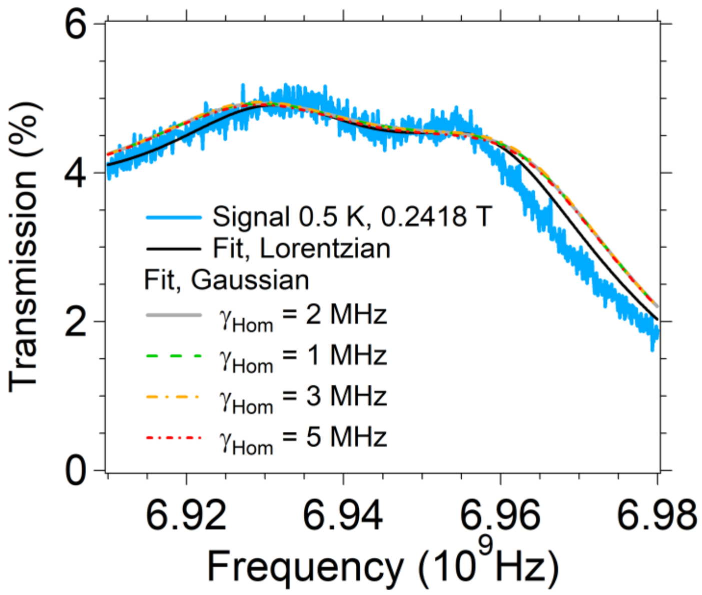

Figure S10 Comparison between the best fits of the data in Fig. 2d (main paper) performed with equation (1) of the main paper (Lorentzian) and with equation (S3) (Gaussian). Solid line represents the best fit obtained for $\gamma_{hom} = 2$ MHz, while the dashed lines are the best fits obtained for $\gamma_{hom} = 1$ MHz, $\gamma_{hom} = 3$ MHz and $\gamma_{hom} = 5$ MHz.

γ_{hom} (MHz)	Δ (MHz)	Ω (MHz)
2	28.5± 3.0	18 ± 2
5	27 ± 3	18.5 ± 2.0
3	27.5 ± 3.0	18 ± 2
1	29 ± 3	18± 3

Table S2. Δ and Ω values obtained from the best fit of the data of Figure 1.f and S10 by means of equation (S3) for different choices of γ_{hom} between 0.5 and 5 MHz (see spin linewidth section).

Since also equation (S3) can qualitatively reproduce the experimental data, some hints can be deduced from the fitting parameters of Table S2. The fitted coupling strength for γ_{hom} = 2 MHz is Ω = 18 ± 2 MHz, which agrees with the result given in the main text (Ω = 21 ± 3 MHz). Moreover we have Δ = 28.5 ± 3 MHz, in agreement with the γ obtained with the Lorentzian curve (γ = 30 ± 4 MHz). This confirms that the values of γ given in the main text are dominated by the inhomogeneous broadening contributions and correspond to an upper limit for the spin linewidth (for our set-up) and therefore, the corresponding C = 15 constitute a lower bound for the cooperativity. Conclusions similar to the previous ones can be asserted also for the different choices of γ_{hom} between 5 MHz and 0.5 MHz (Table S2).

CURVE FITTING OF $\delta\nu/\nu_0$ vs B_0 IN THE WEAK COUPLING REGIME

Within the framework of a Lumped Element theory, the resonator can effectively be treated as a classical RLC oscillator, while the spin ensemble can be modelled as an inductance and a resistance that depend on the dynamic magnetic susceptibility of the ensemble [12,13,14]. By generalizing the case of a single ensemble, in which the same Ω and γ parameters of equation (1) can be introduced (see main paper) the multi-folded signal is modelled as a set of independent spin ensembles coupled to the cavity mode, each one characterized by its coupling rate, Ω_j, linewidth, γ_j, and transition energy,$\nu_{S,j}$ [14] (equation S4).

$$\nu = \nu_0 + \sum_{j=1}^{\#lines} \frac{\Omega_j^2(\nu_0 - \nu_{S,j})}{(\nu_0 - \nu_{S,j})^2 + \gamma_j^2}$$

(S4)

In this way, equation (S4) is used to fit all the $\delta\nu/\nu_0$ vs B_0 data in the weak coupling regime, giving the values of Ω and γ for the main line of Figure 3.a of the main paper. A set of best fit in the weak coupling regime is given in Figure S11.

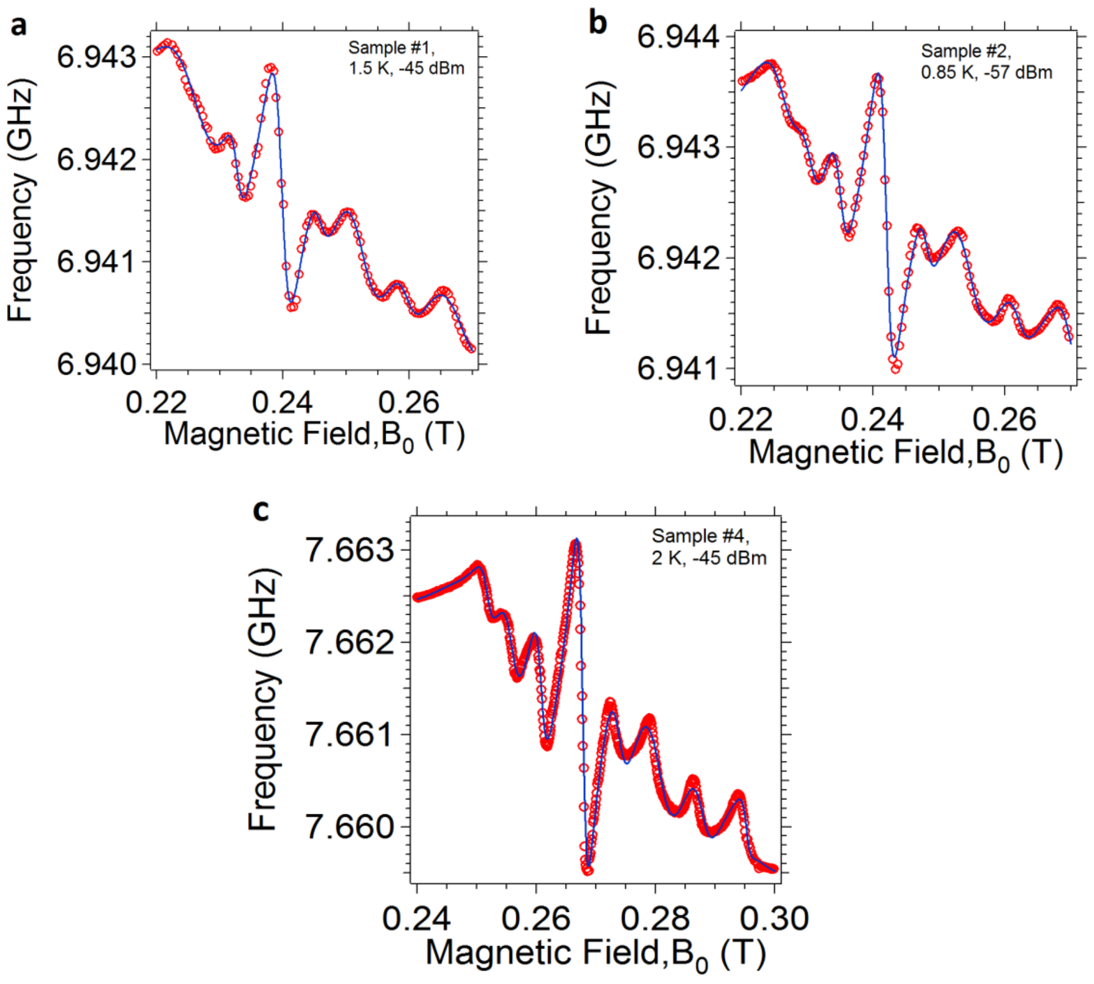

Figure S11 Fitting of the resonant frequency as a function of the magnetic field in the weak coupling regime performed with equation S4 for sample #1 (a), Sample #2 (b) and Sample #4 (c).

ELECTROMAGNETIC SIMULATION

Derivation of equation (2) of the main paper. Within the Lumped Element theory, the coupling rate for a given jth ensemble is calculated starting from equation (S5) [15]. Here, B_1 is the magnetic field generated by a single photon in the cavity, g is the Landè g-factor (we assume the Landè g-tensor to be isotropic), n is the room temperature spin density of the sample, V is the sample volume and $|G_i\rangle$, $|E_i\rangle$ are the states involved in the transition.

$$\Omega_j = g\frac{\mu_B}{h}\sqrt{np_j(T)\int_V \left|\langle G_j|\,\vec{B}_1\cdot\vec{S}|E_j\rangle\right|^2 dV}$$

(S5)

In a similar way to what is done in ref [15,16], equation (S5) can be simplified by exploiting the symmetry of the resonant mode respect to the axis of the resonator. This allows to separate the magnetic component from the transition matrix element of equation (S5), providing that $\left|\langle G_j|\,\vec{B}_1\cdot\vec{S}|E_j\rangle\right|^2 = \left|\langle G_j|\,S_\perp|E_j\rangle\right|^2|B_1|^2$ (being $S_\perp$ the projection of the spin operator along the direction perpendicular to $\vec{B}_0$). This way, the integration can be performed only on the magnetic field. If the maximum magnetic fields for the cavity populated by a single photon, $B_{1,max}$, is introduced in equation (S5) we have

$$\Omega_j = \frac{g\mu_B}{h}\left|\langle G_j|\,S_\perp|E_j\rangle\right|\left|B_{1,max}\right|\sqrt{np_j(T)\int_V |\beta|^2 dV}$$

where $\beta = B_1/B_{1,max}$ is the 3D profile of the magnetic field and correspond to the spatial distribution of the field. To arrive at equation (2) of the main text we define:

$$\Omega_{s,j} = \frac{g\mu_B}{h}\left|\langle G_j|\,S_\perp|E_j\rangle\right|\left|B_{1,max}\right|$$

(S6.a)

$$N_{eff,j} = np_j(T)\int_V |\beta|^2 dV = p_j(T)N_0$$

(S6.b)

$$N_0 = n\int_V |\beta|^2 dV$$

(S6.c)

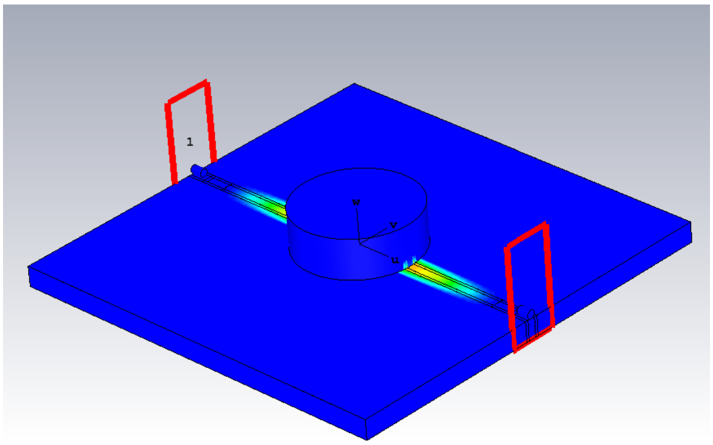

Figure S12 Sketch of the 3D model of the resonator for the electromagnetic simulation. The cylinder in the middle represents the volume of the pellet. The magnetic field of the fundamental resonant mode is plotted (rainbow scale, arbitrary units).

Simulation of the number of spins. Finite-element simulations of the microwave electromagnetic field are performed with the software *CST Microwave Studio*. A 3D 1:1 scale model of the resonator is designed, and the pellet is represented by a cylindrical solid with $\varepsilon_r = 1$, as in Figure S12. The dimension of the pellets are estimated under optical microscope (Table S3). The integral in equations S6, are directly calculated by the software's tools (Table S3). By assuming $p(T) = \tanh\left(\frac{h\nu_0}{2k_BT}\right)$ (see main paper), equation S6.b is used to predict the effective number of spin for a given temperature (dashed lines of Figure 3.c of the main paper). Our simulations give $B_{1,max} \approx 6 \cdot 10^{-11}$ T for Res #1, and $B_{1,max} \approx 3 \cdot 10^{-12}$ T for Res #2.

Sample	∅ (mm)	h(mm)	$\int_V \lvert\beta\rvert^2 dV$ (m^3)
#1	3	1	$7.1 \cdot 10^{-9}$
#2	3	1	$7.1 \cdot 10^{-9}$
#3	5	0.5	$7.8 \cdot 10^{-9}$
#4	5	0.3	$2.9 \cdot 10^{-9}$
#5	3	1	$7.1 \cdot 10^{-9}$

Table S3. Dimensions of the pellets used in our experiments and corresponding quantities of equations (S6.c) given by the electromagnetic simulations. For sample #4 only half of the pellet was placed on the resonator.

Estimation of the transition matrix elements. In principle, equation S6.a could be also used to predict the single spin coupling, since $B_{1,max}$ is known from the simulation. A rough estimation could be done by considering that a pure S=1/2 spin with no anisotropy would give $|\langle G_j| \vec{S}|E_j\rangle| = \sqrt{2}/2$ [15,16]. It is also known that hyperfine splitting in the energy spectrum have the effect to reduce Ω_s respect to the S=1/2, I=0 case [7]. Since we work at temperature above 300 mK, we can assume that the energy levels are equally populated and, hence, that the single spin coupling expected for a pure S=1/2 system is equally distributed among the eight transitions. This allows to estimate the matrix element as $|\langle G_j| \vec{S}|E_j\rangle| \approx 1/8 \cdot \sqrt{2}/2 = \sqrt{2}/16$ for each j. This is confirmed also by considering that, for Res #2 and a pure spin ½ (DPPH), $\Omega_{s,s=1/2} = 0.5$ Hz [4] and in this work we have $\Omega_s = \frac{0.5}{8} \approx$ 0.06 Hz (for sample #4). This value corroborates the $\Omega_{s,fit}$ results of Table 1 of the main paper. Similar arguments were found to hold also in [7]. In fact, the $Cu(mnt)_2$ sample (S=1/2, I=3/2) had $\Omega_s = 0.1\ Hz$, which is in good agreement with $\Omega_s = \frac{\Omega_{S,S=1/2}}{(2*\frac{3}{2}+1)} = \frac{0.5}{4} \approx 0.12\ Hz$.

FITTING OF Ω-T DEPENDENCE.

Figure S13 reports the fitted Ω as a function of the temperature for all samples. Dashed lines are the fits based on equation (2) of the main paper.

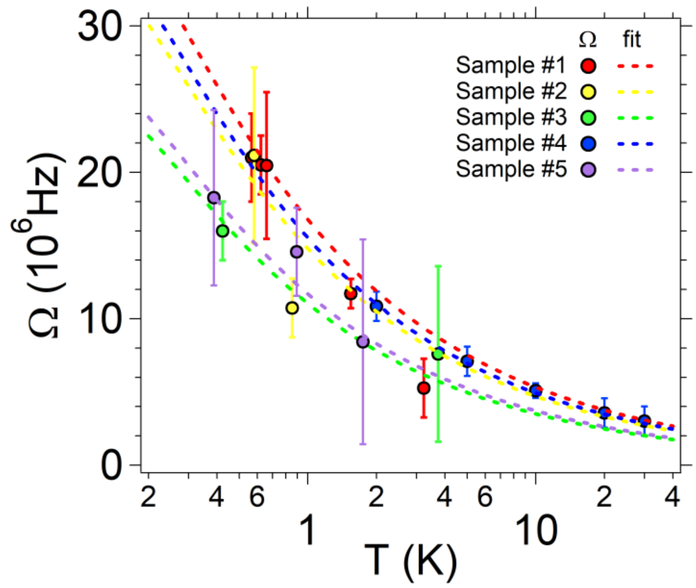

Figure S13 Coupling rates as a function of the temperature for all the 10% and 30% samples. Dashed lines are the fits according to equation (2) of the main paper.

ERRORS AND UNCERTAINTIES ESTIMATION

Errors on the bare Q factors. Errors bars on the Q factors of Table S1 and Figure S5 take into account of the reproducibility of the best fit values over the different choices of the models used to checking their consistency (Equation (1) of the main paper, model from [4] and "-3 dB rule" [6], see resonator section).

Errors on the best fit of *Ω, γ and Δ* parameters. Errors on Ω and on the linewidths (γ of Equation (1) of the main paper or Δ of Equation (S3)) are estimated by checking the robustness and the reproducibility of the values when the initialization of the fitting parameters is changed.

Errors on the number of spins. The simulation of N_0 requires the measure of sample volume, which is done under optical microscope; the relative error on the sample error has

been quantified in 10%. In the simulations, samples are assumed to be perfectly on contact with the surface of the resonator, without interstitial spaces or gaps. Because of the roughness of the pellets, this could lead to an overestimation of the simulated N_0, as in sample #3. Note that sample #3 is the pellet with the highest diameter (Table S3) and it's reasonable to consider heavier effects from the variations of the contact area. Errors on the fitted N_0 and Ω_S are estimated as done for Ω and γ. Finally, the errors on the simulated Ω_S has been calculated with standard error propagation.

REFERENCES

[1] Atzori, M. et al. Room-temperature quantum coherence and rabi oscillations in vanadyl phthalocyanine: toward multifunctional molecular spin qubits. *J. Am. Chem. Soc.* **138**, 2154-2157 (2016).

[2] Stoll, S. & Schweiger, A. Easyspin, a comprehensive software package for spectral simulation and analysis in epr. *Journ. Magn. Reson.* **178**, 42-55 (2006).

[3] Atzori, M. et al. Quantum coherence times enhancement in vanadium(iv)-based potential molecular qubits: the key role of the vanadyl moiety. *J. Am. Chem. Soc.* ***138***, 11234–11244 (2016).

[4] Ghirri, A. et al. $YBa_2Cu_3O_7$ microwave resonators for strong collective coupling with spin ensembles. *Appl. Phys. Lett.* **106**, 184101 (2015).

[5] Kanaya, H. & Fujiyama, J. & Oba, R. & Yoshida, K. Design method of miniaturized HTS coplanar waveguide bandpass filters using cross coupling. *Appl. Supercond., IEEE Transactions on* **13**, 265-268 (2003).

[6] Pozar, D. M. Microwave Engineering. (John Wiley & Sons, 2012).

[7] Bonizzoni, C. et al. Coupling molecular spin centers to microwave planar resonators: towards integration of molecular qubits in quantum circuits. *Dalton Trans.* **45**, 16596-16603 (2016).

[8] Weil, J. A. & Bolton, J. R. Electron Paramagnetic Resonance, elementar theory and applications (John Wiley & Sons, Hoboken, New Jersey, 2007).

[9] A. Schweiger & G. Jeschke. Principles of Pulse Electron Paramagnetic Resonance. Oxford University Press. (Oxford, 2001).

[10] Ghirri, A. et al. Coherently coupling distinct spin ensembles through a high-Tc superconducting resonator. *Phys. Rev. A* **93**, 063855 (2016).

[11] Diniz, I. et al. Strongly coupling a cavity to inhomogeneous ensembles of emitters: Potential for long-lived solid-state quantum memories. *Phys. Rev. A* **84**, 063810 (2011).

[12] Bushev, P. et al. Ultralow-power spectroscopy of a rare-earth spin ensemble using a superconducting resonator. *Phys. Rev. B* **84**, 060501 (2011).

[13] Abragam, A. & Bleaney, B. Electron Paramagnetic Resonance of Transition Ions. (Oxford Classics Texts in the Physical Sciences, Oxford, 2012).

[14] Boero, G. et al. Room temperature strong coupling between a microwave oscillator and an ensemble of electron spins. *J. of Magn. Res.* **231**, 133-140 (2013).

[15] Jenkins Sanchez, M. D. et al. Coupling single-molecule magnets to quantum circuits. *New Journ. of Phys.* **15**, 095007 (2013).

[16] Jenkins Sanchez, M. D. Coupling quantum circuits to magnetic molecular qubits. PhD Thesis, Prensas dela Universidad, Universidad Zaragoza (2015).
